# The impact of the COVID-19 outbreak on emergency general surgery in the first German “hotspot region” Aachen-Heinsberg–A multicentre retrospective cohort study

**DOI:** 10.1371/journal.pone.0280867

**Published:** 2023-01-25

**Authors:** Alexandros Chrysos, Iakovos Amygdalos, Priscila Nunes, Guenter Haselow, Konstantinos Lioupis, Raphael Rosch, Roman Marius Eickhoff, Georg Wiltberger, Ulf Peter Neumann, Andreas Lambertz

**Affiliations:** 1 Department of General, Visceral and Transplantation Surgery, RWTH University Hospital, Aachen, Nordrhein-Westfalen, Germany; 2 Department of General Surgery, St. Elisabeth-Hospital, Geilenkirchen, Nordrhein-Westfalen, Germany; 3 Department of General Surgery, Municipal Hospital, Heinsberg, Nordrhein-Westfalen, Germany; 4 Department of General Surgery, Hermann-Josef-Hospital, Erkelenz, Nordrhein-Westfalen, Germany; Stanford University School of Medicine, UNITED STATES

## Abstract

This study aimed to observe the impact of the COVID-19 outbreak on acute general surgery in the first German “hotspot” regions of Heinsberg and Aachen, during the first months of the pandemic. The incidence and severity of acute appendicitis, acute cholecystitis and mechanical bowel obstruction, were compared between March and May 2020 and a control period (same months of the previous three years). Pre-, intra- and postoperative data was compared between three regional hospitals of Heinsberg and the closest maximum care, university hospital. A total of 592 operated patients were included, 141 belonging to the pandemic cohort and 451 to the historic cohort. The pandemic group showed higher rates of clinical peritonitis (38% vs. 27%, p = 0.015), higher rates of mean white blood cell count (13.2±4.4 /nl vs. 12.3±4.7 /nl, p = 0.044) and mean C-reactive protein (60.3±81.1 mg/l vs. 44.4±72.6 mg/l, p = 0.015) preoperatively. Specifically in patients with acute appendicitis, there were less patients with catarrhal appendicitis (23% vs. 35%, p = 0.021) and a tendency towards more advanced histological findings in the pandemic cohort. In the university hospital, a 42% reduction in acute operated cases was observed at the onset of the pandemic (n = 30 in 2020 vs. n = 52 in 2019), whereas in the peripheral hospitals of Heinsberg there was only a 10% reduction (n = 111 in 2020 vs. n = 123 in 2019). The onset of the COVID-19 pandemic in our region was accompanied by advanced preoperative and intraoperative findings in patients undergoing emergency general surgery. A greater reduction in acute operated surgical cases was observed at the university hospital, in contrast to the smaller hospitals of Heinsberg, suggesting a possible shift of emergency patients, requiring immediate operation, from maximum care hospital to the periphery.

## Introduction

In December 2019, a rapidly spreading pneumonia outbreak of unknown origin appeared in Wuhan, China, characterized by fever, cough, fatigue and occasional gastroenterological symptoms. The causative pathogen was later identified as the SARS-CoV-2 virus and the World Health Organization defined the infectious disease caused by it as COVID-19 (coronavirus disease 2019) [1–4].

The first infection in Germany was confirmed on January 27^th^, 2020 [5], followed by a cluster of COVID-19 cases in the region of Heinsberg, which became the first German “hotspot” [6]. Despite containment measures, the virus spread from Heinsberg to neighbouring regions. Many of those patients were treated in the nearest University Hospital (UH), a maximum care hospital. The number of COVID-19 cases rose steadily, both in Germany and worldwide, leading to the implementation of severe infection control strategies, also known as ‘lockdowns’, in various federal states of Germany.

Soon after the first lockdown began, a decrease in new surgical patients in the emergency department of the UH was observed. This often seemed to be combined with advanced clinical findings. This tendency did not appear to be isolated in the region of Aachen and Heinsberg, as seen in the literature [7, 8]. This reduction was mostly observed in the university surgical department between March and May 2020. In order to better understand this, we chose to study the incidence and gravity of the most frequent acute surgical diagnoses in our emergency department, namely acute appendicitis (AA), acute cholecystitis (AC) and mechanical bowel obstruction (MBO), during those three months and compare them to those of the three regional hospitals of Heinsberg [9, 10]. An important aspect of our study was to observe incidence tendencies in surgical clinics of different range and capacity, varying from peripheral, regional, up to a maximum care university clinic.

## Methods

Patients with AA, AC and MBO, who presented in the emergency departments of UH and three peripheral hospitals of the Heinsberg region (PHHR) and were operated between March and May 2020 (“pandemic cohort”) or the same months of the preceding three years (March-May 2017–2019, “historic cohort”), were included in this study. Patients treated conservatively or those presenting with complications, requiring surgical treatment, during hospital stay (for example postoperative MBO) were excluded. Eligible patients were identified from digital healthcare records of all four hospitals, after searching for the relevant International Statistical Classification of Diseases and Related Health Problems (ICD) codes (K35.2-K35.3 for AA, K81.0 for AC, K56.1 –K56.7 for MBO) and German operation and procedure key (OPS) codes (5–470 for appendectomy, 5–511 for cholecystectomy, 5–469 for ileus operations) [11]. Specifically concerning patients with AA, an aggressive operative approach was established long before and maintained during the onset of the pandemic in all four involved clinics. Therefore any patient with suspicion of AA underwent a diagnostic laparoscopy and appendectomy, as long as other differential diagnoses were excluded.

The rate of preoperative (clinical) and intraoperative peritonitis were identified as this study’s primary outcomes. Clinical peritonitis was defined as acute abdominal pain with rigid abdomen and/or muscular guarding of the abdomen. Intraoperative peritonitis was defined as redness of the peritoneum, with possible presence of fibrin deposits, pus or faecal matter in the abdominal cavity. The histological grade of inflammation was chosen as a secondary outcome. This study adhered to the Strengthening the Reporting of Observational studies in Epidemiology (STROBE) guidelines [12]. The two groups were compared according to preoperative, intraoperative and postoperative parameters. Patient age, sex, body mass index (BMI), co-morbidities, clinical signs of peritonitis, white blood cell counts (WBC) and C-reactive protein (CRP) levels in serum were recorded at the point of first examination in the emergency department. Intraoperative parameters consisted of operation type and duration, peritonitis, Mannheim peritonitis index (MPI), hollow organ perforation and histological findings. Histological findings of patients with AA and AC were graded according to severity. A score of 1–4 was assigned to histological findings of a catarrhal, phlegmonous, gangrenous and perforated appendicitis respectively. In AC, chronic cholecystitis was scored as 1, ulcerated or phlegmonous cholecystitis as 2, gangrenous as 3 and perforated as 4. We compared the mean values of those scores between the two groups. Postoperative complications were stratified according to the Clavien-Dindo (CD) classification and the comprehensive complication index (CCI) [13, 14]. Furthermore, the frequency of postoperative revisions, intensive care unit (ICU) and hospital length of stay (LOS) were compared between the two groups. We repeated the analysis of the aforementioned parameters in the subgroups corresponding to each diagnosis.

With regards to antibiotic treatment, all clinics followed a standardized protocol, allowing for substitutions in cases of allergies. A single shot of Cefuroxim and Metronidazol was administered before incision. In cases with a localized peritonitis or advanced findings, for instance ulcerated, phlegmonous or perforated cholecystitis and appendicitis, the antibiotic treatment with Cefuroxim and Metronidazol was continued for 3 to 5 days postoperatively according to symptoms and laboratory parameters. In case of deterioration, the antibiotic treatment was escalated to Piperacillin/Tazobactam. There was no difference in treatment between the four surgical departments over those four years. All clinics followed standardised treatment algorithms, with minimal variation, according to German and European guidelines for the aforementioned illnesses. This was verified with all departmental heads before the study started. The four surgical clinics have a longstanding mutual cooperation. The onset of the pandemic was addressed as a regional problem, one which the hospitals tried to overcome united.

After the appearance of the SARS-CoV-2 Virus in Germany, every patient presenting in the emergency department, regardless of diagnosis, received a COVID-19 Polymerase Chain Reaction (PCR) Test. Surgical patients that showed typical COVID-19 symptoms were isolated and treated as possible COVID-19 cases with a simultaneous surgical pathology, until receiving the test results. A negative PCR test result would mean the termination of the isolation procedure. In case of infection confirmation, the diagnosis and treatment algorithm was continued under patient’s isolation and personnel protection measures. In our pandemic cohort, there was no patient with a positive SARS-Cov-2 virus PCR Test.

### Statistical analysis

Statistical analysis was performed using SPSS Statistics v26 (IBM Corp., Armonk, NY, USA) and graphs were generated using Prism v8.0 (GraphPad Software, La Jolla, CA, USA). Categorical data were compared with Pearson’s chi-squared test. Group comparisons were carried out using the Mann-Whitney U test, the Chi-square test or Fisher’s exact test. All *p-*values <0.05 were considered statistically significant. Continuous data are represented as mean ± standard deviation.

### Ethical board approval

The study was conducted under ethical approval of the Institutional Review Board of the RWTH Aachen University (EK-340/20) and in accordance with the current version of the Declaration of Helsinki, the Declaration of Istanbul, and good clinical practice guidelines (ICHGCP). Informed consent was waived due to the retrospective study design and collection of readily available clinical data.

## Results

A total of 592 operated patients (51% female, 48% male) were included in our study, of which 141 (24%) were in the pandemic cohort. Mean age was 41±24 years and 79% of patients were adults. As seen in Table 1, no significant demographic differences were observed between pandemic and historic cohorts.

**Table 1:** All diagnoses.

All diagnoses	All Patients	Historic Cohort	Pandemic Cohort	*p*-value
**Nr.**	592	451 (76%)	141(24%)	
**Age (y)**	41±24	41±25	41±24	0.845
**ASA Score**	2±1	2±1	2±1	0.628
**Diagnosis**				
*Appendicitis*	395 (67%)	298 (66%)	97 (69%)	
*Cholecystitis*	79 (13%)	54 (12%)	25 (18%)	**0.036**
*Bowel Obstruction*	118 (20%)	99 (22%)	19 (13%)	
**Clinical peritonitis**	160 (27%)	111 (27%)	49 (38%)	**0.015**
**WBC (/nl)**	12.5±4.6	12.3±4.7	13.2±4.4	**0.044**
**CRP (mg/l)**	48.2±75	44.4±72.6	60.3±81.1	**0.015**
**Op. Procedure**				
*Laparoscopic*	457 (77%)	339 (75%)	118 (83%)	
*Open*	98 (17%)	83 (19%)	15 (11%)	0.081
*Converted*	37 (6%)	29 (6%)	8 (6%)	
**Operative Duration (min)**	70 ± 51	71 ± 53	66 ± 41	0.860
**Intraoperative Peritonitis**	149 (25%)	103 (23%)	46 (33%)	0.057
**MPI**	3±7	3±6	4±8	**0.037**
**Perforation**				
*no perforation*	512 (87%)	393 (87%)	119 (84%)	
*contained*	76 (12%)	56 (12%)	20 (14%)	0.393
*free*	4 (1%)	2 (1%)	2 (2%)	
**Revision**	34 (6%)	31 (7%)	3 (2%)	**0.035**
**Any Complication**	262 (44%)	209 (46%)	53 (38%)	0.074
**CD 0**	328 (56%)	241 (54%)	87 (62%)	0.212
**CD1**	108 (18%)	80 (18%)	28 (20%)	
**CD2**	79 (13%)	65 (14%)	14 (10%)	
**CD3a**	15 (3%)	11 (2%)	4 (3%)	
**CD3b**	30 (5%)	27 (6%)	3 (2%)	
**CD4a**	7 (1%)	6 (1%)	1 (1%)	**0.020**
**CD4b**	7 (1%)	5 (1%)	2 (1%)	
**CD5**	16 (3%)	15 (3%)	1 (1%)	
**CD≥3b**	60 (10%)	53 (12%)	7 (5%)	
**CCI**	13±23	14±24	10±18	**0.037**
**ICU LOS (d)**	0.8±3	0.9±3.4	0.3±0.8	**0.043**
**General LOS (d)**	5±7.4	5.2±7.8	4.2±5.9	0.242

Values given as mean ± standard deviation or absolute and relative frequencies; Abbreviations used: BMI, Body-Mass-Index; ASA, American society of anaesthesiologists score, WBC, White blood cell count; CRP, C-reactive protein; MPI, Mannheim peritonitis index score; CD, Clavien-Dindo score; CD≥3b, CD score equal or higher than 3b (severe complications), CCI, comprehensive complication index; ICU LOS, intensive care unit length of stay; LOS, Length of stay.

Significantly fewer patients presented with MBO (13% vs 22%, p = 0.036) and more patients had clinical signs of peritonitis (38% vs. 27%, p = 0.015) in the pandemic cohort. Furthermore, both mean WBC count (13.2±4.4 /nl vs. 12.3±4.7 /nl, p = 0.044) and mean CRP levels (60.3±81.1 mg/l vs. 44.4±72.6 mg/l, p = 0.015) were higher in that cohort, as was the mean MPI (4±8 vs. 3±6, p = 0.037). The pandemic cohort also demonstrated fewer severe (CD≥3b) complications (5% vs. 12%, p = 0.020) and a lower mean CCI score (10±18 vs. 14±24, p = 0.037). The mean hospital-LOS was similar in the two groups, but the mean ICU-LOS was significantly lower in the pandemic group (0.3±0.8 vs. 0.9±3.4, p = 0.043). Further perioperative data are outlined in Table 1.

### Acute appendicitis

Of 395 patients in the AA group, 97 (25%) belonged to the pandemic cohort. These patients exhibited increased rates of clinical (44% vs. 31%, p = 0.036) and intraoperative (43% vs. 31%, p = 0.041) peritonitis. Mean WBC count (13.6±4.3 /nl vs. 12.5±4.4 /nl, p = 0.034) and CRP (50.9±70.7 mg/l vs. 37.9±59.5 mg/l, p = 0.048) were also higher in this group. Finally, the mean histology score was higher in the pandemic group (2.2±1.1 vs. 1.8±1.1, p = 0.002) and there were significantly fewer histological findings of catarrhal appendicitis in these patients (23% vs. 35%, p = 0.021). Although the preoperative and intraoperative findings of patients with AA in our study were more advanced during the onset of the pandemic, the rate of major complications did not show any significant difference in the two cohorts (CD≥3b, 2% vs. 4%, p = 0.301). A detailed comparison of AA patients can be found in Table 2.

**Table 2:** Acute appendicitis.

Acute Appendicitis	All Patients	Historic Cohort	Pandemic Cohort	*p-*value
**Nr.**	395	298 (75%)	97 (25%)	0.550
**Age (y)**	30±19	30±19	29±17	0.696
**ASA score**	2±1	2±1	1±1	0.741
**Clinical peritonitis**	125 (32%)	87 (31%)	38 (44%)	**0.036**
**WBC (/nl)**	12.8±4.4	12.5±4.4	13.6±4.3	**0.034**
**CRP (mg/l)**	41.2±62.6	37.9±59.5	50.9±70.7	**0.048**
**Op. Procedure**				
*Laparoscopic*	380 (96%)	286 (96%)	94 (97%)	
*Open*	9 (2%)	8 (3%)	1 (1%)	0.567
*Converted*	6 (2%)	4 (1%)	2 (2%)	
**Operative Duration (min)**	53±28	53±29	52±24	0.958
**Intraoperative Peritonitis**	133 (34%)	92 (31%)	41 (43%)	**0.041**
**MPI**	4±7	3±7	5±8	0.095
**Histology Appendicitis**				
*catarrhal*	127 (32%)	105 (35%)	22 (23%)	**0.021**
*phlegm*	171 (43%)	125 (42%)	46 (47%)	0.344
*gangrenous*	23 (6%)	14 (5%)	9 (9%)	0.094
*perforated*	56 (14%)	38 (13%)	18 (19%)	0.155
*Other*	18 (5%)	16 (5%)	2 (2%)	0.175
**Histology Score**	1.9±1.1	1.8±1.1	2.2±1.1	**0.002**
**Perforation**				
*no perforation*	336 (85%)	258 (86.7%)	78 (80%)	
*contained*	56 (14%)	39 (13%)	17 (18%)	0.122
*free*	3 (1%)	1 (0.3%)	2 (2%)	
**Any Complication**	113 (29%)	91 (31%)	22 (23%)	0.088
**CD≥3b**	15 (3%)	13 (4%)	2 (2%)	0.301
**CCI**	6±13	7±13	5±11	0.119
**ICU LOS (d)**	0.07±0.4	0.07±0.4	0.04±0.2	0.497
**General LOS (d)**	2.9±3	2.8±2.6	3.2±4	0.681

Values given as mean ± standard deviation or absolute and relative frequencies; Abbreviations used: BMI, Body-Mass-Index; ASA, American society of anaesthesiologists score, WBC, White blood cell count; CRP, C-reactive protein; MPI, Mannheim peritonitis index score; CD≥3b, Clavien Dindo score equal or higher than 3b (severe complications), CCI, comprehensive complication index; ICU LOS, intensive care unit length of stay; LOS, Length of stay.

### Acute cholecystitis

In the AC group, no statistically significant differences were observed between cohorts. Detailed information can be found in S1 Table of the Supporting information.

### Mechanical bowel obstruction

In patients with MBO, 19 (16%) were operated during the pandemic period. In this group, significantly fewer primary anastomoses were carried out after intestinal resection (5% vs. 26%, p = 0.046). Interestingly, none of these patients presented with inflamed bowel, in contrast to the historic cohort (0% vs. 18%, p = 0.043). Comparisons between MBO cohorts are outlined in S2 Table of the Supporting information.

### Yearly incidences

**Fig 1:**
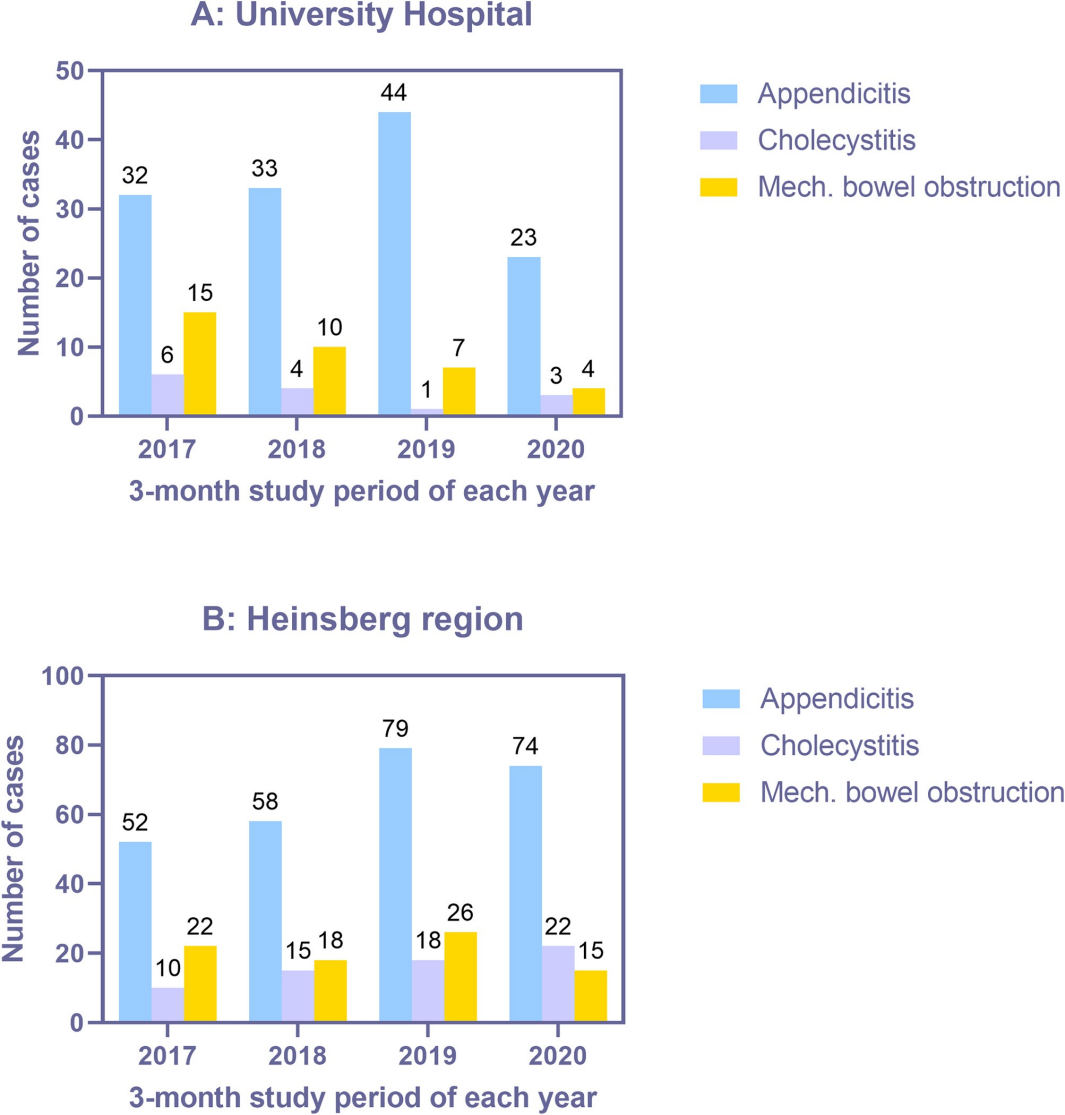
Incidence distribution over the years during the respective three month study intervals. A. University Hospital. B. Heinsberg region.

The overall incidence of AA, AC and MBO during the corresponding study periods of 2017 to 2020 is depicted in Fig 1. Fig 1A depicts the aforementioned incidences in the UH and Fig 1B the incidences in the PHHR. As can be seen in Fig 1A, there was a 48% decline in AA patients and a 43% decline in patients with MBO at the UH, in comparison to 2019. On the contrary, AC cases rose. On the other hand, only MBO cases declined in PHHR (42% reduction in 2020), whereas AA cases remained almost the same, as depicted in Fig 1B. Interestingly, the incidence of AC rose here too.

### Discussion

The COVID-19 pandemic radically changed the regularity of surgical practice. In Germany, as well as other European countries, the sudden need for more ICU beds lead to a severe reduction in elective surgical cases [15]. In the field of general surgery, mostly patients with malignant disease were operated. Moreover, most of the ICU beds were allocated to the treatment of critical COVID-19 patients, leaving little resources for the non-COVID-19 emergencies or oncological patients. Surgeons, anaesthesiologists and physicians of other specialties with ICU experience were transferred to the ICU wards to cover for personnel shortages. Some operating theatres, in areas most struck by the pandemic, were converted to ICU beds to maximize the intensive care capacity of hospitals [16]. Blood products were also becoming more scarce, since donating was drastically reduced through lockdown measures [17]. Laparoscopy, which plays a major role in emergency and elective general surgery, was associated with an increased risk of SARS-CoV-2 virus exposure for the operating personnel, leading to restrictions in its use [18]. All these sudden changes forced surgeons to adapt to new conditions, change their operative workflow and reprioritize, in order to maintain high surgical standards [19]. New measures were implemented to hinder viral spread, while the treatment of oncological and emergency patients had to be guaranteed [20, 21]. As the changes in day-to-day surgery were universal and there was no clinic left unscathed by the pandemic, we included clinics of different capacities.

This study showed an overall decrease in acute operated cases at a tertiary centre (UH), during the study period. This agrees with the results of a Spanish multicentre retrospective study, where a 58% decrease in acute care surgery was observed, as well as a decrease in AC cases between March and April 2020 [22]. In contrast to our study, the number of patients suffering from MBO was statistically higher in 2020, compared to previous years. Another interesting point was the increased time between onset of symptoms and patient arrival at the emergency department during the study period [22]. We believe that the uncertainty of the public during the pandemic was materialized in both studies. The hospitals which were functioning as main COVID-19 institutions were possibly met with hesitation by the public, thus the reduction in AA and MBO patients in the UH. Examples of patients or caregivers avoiding hospitals have also been monitored and published in an Italian paediatric article from Lazzerini et al [23], where parents reported avoiding hospitals for fear of a SARS-CoV-2 viral infection, with grave consequences for some of the children.

In our study, the rate of minor complications was similar among the two cohorts as seen in Table 1. However, the number of severe (CD≥3b) complications and the median CCI were significantly lower in the pandemic group. In contrast to our study, Cano-Valdemarra O. et al suggest, that minor complications were more common during the pandemic because of delayed clinical presentation and advanced intraoperative findings, but severe complications were probably similar in both groups [22]. Our pandemic cohort contained the lowest number of patients with MBO, compared to previous years. These patients are often older, with more comorbidities, higher ASA score, require a more extensive operative treatment and tend to require a longer ICU treatment than those with AA and AC. As a result, MBO patients are more prone to major complications, have a higher CD Score and CCI. We believe the reduced number of MBO patients in the pandemic cohort correlates with the reduced rate of major complications (CD≥3b) and lower median CCI of this cohort. Moreover, ICU LOS was also significantly shorter in our pandemic cohort. This could be attributed to the scarcity of ICU beds and reprioritization of intensive care resources [24]. Surgical patients were probably transferred sooner to intermediate care (IMC) or standard care wards, where the risk of a COVID-19 infection was reduced or were denied ICU beds for less than life-threatening indications.

In our study, the reduced number of catarrhal appendicitis, the increased rate of phlegmonous histology, higher MPI and increased WBC counts and CRP levels in the pandemic AA group demonstrate advanced inflammation in these patients. This coincides with examples from the literature, such as Romero, J. et al, whose results showed fewer appendicitis patients presented in the emergency department, but with a more severe stage of the disease during the pandemic period [25, 26]. The main reason for those findings appears to be the delayed seeking of medical help due to fear of COVID-19 infection. According to Tankel J. et al, another reason for the reduced number of cases during the pandemic could be a successful symptomatic treatment of mild appendicitis at home [27]. Some surgical societies suggested a conservative treatment of uncomplicated appendicitis with antibiotics as a means to limit surgical personnel exposure to COVID-19 aerosol [28, 29]. A reduction of appendicitis patients was observed in our study too, but only in the maximal care clinic. According to the departmental heads of the four clinics involved, a direct operative treatment was favoured in the case of AA. Our study did not investigate the prevalence of conservatively treated cases, as we believe this to be a negligible number, therefore we cannot provide an accurate presentation of conservative treatment.

On the other hand, our study showed a 24% overall increase in cholecystectomy cases during the pandemic. No significant differences were observed between pandemic and historic cohorts, suggesting that the operative treatment and postoperative course of these patients was not severely influenced by the onset of the pandemic.

As mentioned previously, MBO cases declined mostly in the tertiary centre, during the pandemic period of our study. There have been many reports concerning the impact of the pandemic on the incidence of MBO. In Spain and Italy, there was an increase in surgical interventions for MBO in March 2020 [7, 22]. Additionally, a significant reduction in anastomoses after intestinal resection was observed in the pandemic subgroup of our study. This may signify a more cautious intraoperative approach in an era of limited personnel and resources, as a postoperative anastomotic leak could further siphon the available workforce and prolong the patients’ stay in the intensive care. This cautious approach could also have attributed to the pandemic cohort’s reduced major complications.

The reduction of acute surgical cases at the university hospital during the study period could suggest a public tendency to avoid large hospitals, where COVID-19 patients were primarily treated and where a higher “crowd density” (healthcare personnel and patients) was to be expected during emergency hours. On the other hand, it may also reflect a tendency to move locally and visit the next available hospital, instead of a tertiary centre, which was common practice before, even for clinical presentations that did not warrant it. Additionally, the lack of intensive care beds at the university hospital led to a decrease in emergency patient referrals from the periphery (for example in high-risk patients with cholecystitis and mechanical bowel obstruction). This possible shift of acute surgical patients from the tertiary centre towards the peripheral clinics of Heinsberg at the onset of the pandemic could be a more generalised phenomenon that exceeds the regions of Aachen and Heinsberg.

Our study suffers from some limitations, such as its retrospective nature and the inhomogeneity of three different diagnosis-groups, each with different pre-, intra- and postoperative aspects. Nevertheless, we tried to approach and analyse each group systematically, with similar outcome parameters. As only operated patients were included, we can expect that the general incidence of the aforementioned diagnoses was probably higher. The tendency to a more conservative treatment of non-complicated surgical emergencies during the pandemic should be further explored. Additional prospective studies are required, in order to better understand the ongoing illness tendencies and the full impact of COVID-19 in general surgery.

## Conclusion

We can conclude, that the onset of the COVID-19 pandemic had a considerable impact on emergency gastrointestinal surgery. Specifically, this study demonstrated significant differences in clinical, laboratory, intraoperative and histological findings of patients undergoing appendectomies between historical and pandemic cohorts, pointing towards more advanced disease in the pandemic group. Additionally, a reduction of operated patients was observed in the university hospital during the pandemic period, which was not reflected in the caseload of the peripheral hospitals. These results may suggest a delayed presentation of patients to the emergency department, combined with a shift towards peripheral hospitals. A tendency towards conservative treatment, should also be considered regarding this reduction of operated patients. Further studies are necessary to elucidate the reasons behind these observations.

## Supporting information

S1 Table Acute cholecystitis. (DOCX)

S2 Table Mechanical bowel obstruction table. (DOCX)

S1 Data (SAV)

S2 Data (TXT)

## Abbreviations

AA: Acute appendicitis
AC: Acute cholecystitis
ASA: American society of anaesthesiologists
BMI: Body mass index
CCI: Comprehensive complication index
CD: Clavien—Dindo
COVID-19: Coronavirus disease 2019
CRP: C-reactive protein
ICU: Intensive Care Unit
ICU LOS: Intensive care unit length of stay
LOS: Length of stay
MBO: Mechanical bowel obstruction
MPI: Mannheim peritonitis inde
PCR: Polymerase Chain Reaction
PHHR: Peripheral hospitals of Heinsberg region
UH: University Hospital
WBC: White Blood Cells
