# Supplementary material for: The impact of the COVID-19 outbreak on emergency general surgery in the first German “hotspot region” Aachen-Heinsberg–A multicentre retrospective cohort study
Source: PLoS One. 2023 Jan 25;18(1):e0280867. doi: 10.1371/journal.pone.0280867 (PMC9876361; doi:10.1371/journal.pone.0280867)
Supplement: S1 Table — (DOCX) [file pone.0280867.s001.docx]

**S1 Table. Acute cholecystitis**

| **Acute Cholecystitis** | **All Patients** | **Historic Cohort** | **Pandemic Cohort** | ***p-*value** |
| --- | --- | --- | --- | --- |
| **Nr.** | 79 | 54 (68%) | 25 (32%) |  |
| **Age (y)** | 62±16 | 61±15 | 65±19 | 0.180 |
| **ASA score** | 2±0.3 | 2±1 | 2±1 | 0.471 |
| **Clinical peritonitis** | 19 (24%) | 13 (26%) | 6 (26%) | 0.994 |
| **WBC (/nl)** | 12.5±5.2 | 12.6±5.5 | 12.1±4.7 | 0.971 |
| **CRP (mg/l)** | 99.8±121.5 | 97.9±126.1 | 104.1±113.3 | 0.971 |
| **Op. Procedure**  *Laparoscopic*  *Open*  *Converted* | 64 (81%)  5 (6%)  10 (13%) | 43 (80%)  4 (7%)  7 (13%) | 21 (84%)  1 (4%)  3 (12%) | 0.833 |
| **Operative Duration (min)** | 85±46 | 83±45 | 88±48 | 0.624 |
| **Intraoperative Peritonitis** | 15 (19%) | 10 (19%) | 5 (21%) | 0.870 |
| **MPI** | 4±8 | 3±7 | 5±10 | 0.672 |
| **Histology Cholecystitis**  *Chronic*  *Phlegmonous*  *Gangrenous*  *Perforated* | 22 (28%)  30 (38%)  13 (16%)  14 (18%) | 18 (33%)  18 (34%)  7 (13%)  11 (20%) | 4 (16%)  12 (48%)  6 (24%)  3 (12%) | 0.110  0.212  0.219  0.365 |
| **Histology Score** | 2.2±1.1 | 2.2±1.1 | 2.3±0.9 | 0.457 |
| **Perforation**  *no perforation*  *contained*  *free* | 64 (81%)  14 (18%)  1 (1%) | 42 (78%)  11 (20%)  1 (2%) | 22 (88%)  3 (12%)  0 (0%) | 0.507 |
| **Any Complication** | 41 (52%) | 27 (50%) | 14(58%) | 0.496 |
| **CD≥3b** | 6 (8%) | 6(11%) | 0 (0%) | 0.089 |
| **CCI** | 14±22 | 15±24 | 11±13 | 0.959 |
| **ICU LOS (d)** | 0.5±1.6 | 0.6±1.8 | 0.2±0.6 | 0.959 |
| **General LOS (d)** | 5.9±7 | 6.2±6.9 | 5.3±7.1 | 0.387 |

Legend: Values given as mean ± standard deviation or absolute and relative frequencies; Abbreviations used: BMI, Body-Mass-Index; ASA, American society of anaesthesiologists score, WBC, White blood cell count; CRP, C-reactive protein; MPI, Mannheim peritonitis index score; CD≥3b, Clavien Dindo score equal or higher than 3b (severe complications), CCI, comprehensive complication index; ICU LOS, intensive care unit length of stay; LOS, Length of stay.
