# Supplementary material for: The impact of the COVID-19 outbreak on emergency general surgery in the first German “hotspot region” Aachen-Heinsberg–A multicentre retrospective cohort study
Source: PLoS One. 2023 Jan 25;18(1):e0280867. doi: 10.1371/journal.pone.0280867 (PMC9876361; doi:10.1371/journal.pone.0280867)
Supplement: S2 Table — (DOCX) [file pone.0280867.s002.docx]

**S2 Table. Mechanical Bowel Obstruction Table**

| **Mechanical Bowel Obstruction** | **All Patients** | **Historic Cohort** | **Pandemic Cohort** | ***p-*value** |
| --- | --- | --- | --- | --- |
| **Nr.** | 118 | 99 (84%) | 19 (16%) |  |
| **Age (y)** | 65±19 | 65±19 | 64±201 | 0.936 |
| **ASA Score** | 3±1 | 3±1 | 3±1 | 0.574 |
| **Clinical peritonitis** | 16 (14%) | 11 (13%) | 5 (28%) | 0.120 |
| **WBC (/nl)** | 12.5±9.1 | 12.6±9.7 | 12.4±4.9 | 0.616 |
| **CRP (mg/l)** | 37±56.4 | 34.5±54.7 | 50.4±64.5 | 0.234 |
| **Op. Procedure**  *Laparoscopic*  *Open*  *Converted* | 13 (11%)  84 (71%)  21 (18%) | 10 (10%)  71 (72%)  18 (18%) | 3 (16%)  13 (68%)  3 (16%) | 0.762 |
| **Operative Duration (min)** | 120 ±73 | 121±76 | 117±51 | 0.672 |
| **Intraoperative Peritonitis** | 1 (1%) | 1 (1%) | 0 (0%) | 0.667 |
| **Operative Management**  *Adhesiolysis*  *Open Decomp.*  *Resection*  *Anastomosis*  *Stoma* | 86 (73%)  12 (10%)  44 (37%)  27 (23%)  25 (21%) | 69 (70%)  12 (12%)  40 (40%)  26 (26%)  20 (20%) | 17 (90%)  0 (0%)  4 (21%)  1 (5%)  5 (26%) | 0.076  0.109  0.110  **0.046**  0.550 |
| **MPI** | 0.3±3 | 0.3±3 | 0 | 0.668 |
| **Histology Intestine**  *No Resection*  *Inflamed*  *Ischemic*  *Perforated*  *Other*  *Normal* | 71 (60.2%)  18 (15.3%)  12 (10.2%)  4 (3.4%)  12 (10.2%)  1 (0.8%) | 56 (56.6%)  18 (18.2%)  10 (10.1%)  4 (4%)  10 (10.1%%)  1 (1%) | 15 (78.9%)  0 (0 %)  2 (10.5%)  0 (0 %)  2 (10.5%)  0 (0 %) | 0.068  **0.043**  0.955  0.373  0.955  0.660 |
| **Perforation**  *no perforation*  *contained*  *free* | 112 (95%)  6 (5%)  0 (0%) | 93 (94%)  6 (6%)  0 (0%) | 19 (100%)  0 (0%)  0 (0%) | 0.271 |
| **Any Complication** | 107 (91%) | 90 (91%) | 17 (90%) | 0.844 |
| **CD≥3b** | 39 (33%) | 34 (34%) | 5 (26%) | 0.496 |
| **CCI** | 36±32 | 37±32 | 34±32 | 0.708 |
| **ICU LOS (d)** | 3.3±5.9 | 3.7±6.3 | 1.4±1.6 | 0.190 |
| **General LOS (d)** | 11.1±12.6 | 11.8±13 | 7.7±10.1 | 0.084 |

Legend: Values given as mean ± standard deviation or absolute and relative frequencies; Abbreviations used: BMI, Body-Mass-Index; ASA, American society of anaesthesiologists score, WBC, White blood cell count; CRP, C-reactive protein; MPI, Mannheim peritonitis index score; CD≥3b, Clavien Dindo score equal or higher than 3b (severe complications), CCI, comprehensive complication index; ICU LOS, intensive care unit length of stay; LOS, Length of stay.
